# Genome-Wide Analysis Reveals a Complex Pattern of Genomic Imprinting in Mice

**DOI:** 10.1371/journal.pgen.1000091

**Published:** 2008-06-06

**Authors:** Jason B. Wolf, James M. Cheverud, Charles Roseman, Reinmar Hager

**Affiliations:** 1 Faculty of Life Sciences, University of Manchester, Manchester, United Kingdom; 2 Department of Anatomy and Neurobiology, Washington University School of Medicine, St Louis, Missouri, United States of America; 3 Department of Anthropology, University of Illinois at Urbana-Champaign, Urbana, Illinois, United States of America; University of Cambridge, United Kingdom

## Abstract

Parent-of-origin–dependent gene expression resulting from genomic imprinting plays an important role in modulating complex traits ranging from developmental processes to cognitive abilities and associated disorders. However, while gene-targeting techniques have allowed for the identification of imprinted loci, very little is known about the contribution of imprinting to quantitative variation in complex traits. Most studies, furthermore, assume a simple pattern of imprinting, resulting in either paternal or maternal gene expression; yet, more complex patterns of effects also exist. As a result, the distribution and number of different imprinting patterns across the genome remain largely unexplored. We address these unresolved issues using a genome-wide scan for imprinted quantitative trait loci (*i*QTL) affecting body weight and growth in mice using a novel three-generation design. We identified ten *i*QTL that display much more complex and diverse effect patterns than previously assumed, including four loci with effects similar to the callipyge mutation found in sheep. Three loci display a new phenotypic pattern that we refer to as bipolar dominance, where the two heterozygotes are different from each other while the two homozygotes are identical to each other. Our study furthermore detected a paternally expressed *i*QTL on Chromosome 7 in a region containing a known imprinting cluster with many paternally expressed genes. Surprisingly, the effects of the *i*QTL were mostly restricted to traits expressed after weaning. Our results imply that the quantitative effects of an imprinted allele at a locus depend both on its parent of origin and the allele it is paired with. Our findings also show that the imprinting pattern of a locus can be variable over ontogenetic time and, in contrast to current views, may often be stronger at later stages in life.

## Author Summary

For certain genes, individuals express only the copy of the gene they inherit from either their mother (“maternally expressed” genes) or their father (“paternally expressed” genes). This “parent-of-origin–dependent” pattern of gene expression is known as genomic imprinting and has been shown to play an important role in modulating a variety of traits ranging from developmental processes to cognitive abilities and associated disorders. While various molecular techniques have allowed for the identification of many imprinted genes, very little is known about the contribution of imprinting to variation seen among individuals in continuously varying traits such as body size. Here we address this issue by using a genome-wide analysis aimed at finding regions of the genome that show an effect of imprinting on body weight and growth in mice. We identified ten loci that displayed complex and diverse patterns of effect, including four loci with effects similar to the unusual callipyge mutation found in sheep and three that displayed a new phenotypic pattern that we refer to as bipolar dominance. Surprisingly, most imprinting effects were strongest during the post-weaning period, and many showed shifts in the pattern of imprinting over ontogenetic time.

## Introduction

Genomic imprinting refers to the phenomenon of monoallelic gene expression that depends on the parent-of-origin of alleles, where either the maternally or paternally inherited copy is expressed while the other copy is silenced [1],[2]. The uniparental expression of paternally and maternally derived genes is usually caused by an epigenetic mark of differential methylation set during gametogenesis [2]. Imprinted genes have been shown to crucially affect the development and expression of complex traits such as growth and development [3] throughout life, ranging from early embryonic stages to postnatal and adult phenotypes, and often with tissue specific expression [e.g. 4],[5]. For example, studies have demonstrated that imprinted genes affect cognitive abilities [4],[6], several major human disorders (e.g. Prader-Willi and Angelman syndrome), and possibly obesity [7]–[11]. In the quest to investigate the genetic basis of such complex traits, genome-wide association and linkage studies have become a powerful tool [e.g. 12],[13] where regions of the genome are identified that contain sequence variants associated with phenotypic variation or the presence of a specific disorder. However, very few of such studies have attempted to include investigations of epigenetic variation caused by genomic imprinting despite the known significant effects on complex traits [e.g. 14],[15].

Most of our current knowledge about the number, distribution and effects of imprinted genes comes from studies using gene-targeting techniques focusing on regions of the genome with chromosomal aberrations [1]. Under this methodology, several large-effect imprinted genes and their gross phenotypic effects have been described [e.g. 3],[4]. Most prior studies on imprinting assume the traditional phenotypic pattern resulting from the monoallelic expression of the paternal or maternal allele [e.g. 10],[16]. Yet, more complex patterns exist such as the callipyge phenotype described in sheep, where one of the two heterozygotes shows muscular hypertrophy while the other three genotypes have normal appearance and do not differ from each other [17]. Moreover, other studies demonstrated that loci can deviate from the typical imprinting patterns of uniparental expression where loci may show differential expression of the two parental alleles [e.g. partial expression: 18]. In addition, recent work suggests that many more loci may be imprinted than previously assumed [19]. However, little is known both about the quantitative effects of genomic imprinting and the diversity of patterns of expression.

Furthermore, while both large and small additive and dominance effects have been successfully mapped for a wide variety of traits [20], relatively little empirical research has been conducted into the nature and effects of allelic diversity on quantitative trait variation at imprinted loci. Data on alleles with relatively minor quantitative effects could potentially have important implications for normal physiological and behavioral variation and the expression of complex disease-related traits. Most studies of genomic imprinting have focused on complete knock-outs of a specific locus and, thus, reveal limited information on the effects of different less severe alleles.

Using a three-generational intercross between two inbred strains originally selected for divergent adult body size [LG/J and SM/J; 21], we scanned the genome for loci showing significant parent-of-origin effects on body size and growth traits. We present a hypothetical model for the functional origin of these complex effects and demonstrate that further dissection of imprinted quantitative trait loci (*i*QTL) is likely to yield a more comprehensive understanding of the complex patterns and likely evolutionary origins of imprinting.

## Results

In a genome-wide scan for *i*QTL, we detected ten loci on six chromosomes showing significant parent-of-origin dependent effects that were characterized by a diversity of genomic imprinting patterns (Table 1). Five of these loci exceeded the genome-wide significance threshold and four were significant at the chromosome level. The remaining locus was identified as having an imprinting effect (*i*) that was significant at the chromosome level, but the overall test for the locus was not significant. However, because of the strong parent-of-origin effect at the locus, it was included as a suggestive *i*QTL. Significance tests using the fit of the various possible forms of imprinting (see below) suggest that 8 of the 10 loci were significant at the genome-wide threshold, with the other two being significant at the chromosome level, lending additional support to our findings that these loci represent true *i*QTL. Post-hoc analyses tested whether the parent-of-origin effect appeared in heterozygous offspring of heterozygous mothers and confirmed that the parent-of-origin dependent effects of these ten loci were indeed caused by genomic imprinting and not maternal genetic effects. These analyses revealed that the parent-of-origin dependent effects of five *other* loci were caused by maternal genetic effects, and consequently, these loci are discussed elsewhere [22].

**Table 1:** Imprinted QTL (*i*QTL) with their patterns as defined in the text (see also Figure 1).

	*i*QTL	*Wti1.1*	*Wti2.1*	*Wti3.1*	*Wti3.2*	*Wti3.3*	*Wti5.1*	*Wti 7.1*	*Wti9.1*	*Wti12.1*	*Wti14.1*	*R* ^2^ [%] *i*	*R* ^2^ [%] model
**Location**		8.26	0	1.73	60.71	82.69	82.46	27.7	52.22	42.80	37.75		
**Coordinate**		31.05	3.38	10.02	118.36	148.86	143.58	56.32	99.65	94.76	81.55		
**Confidence interval**		11.04–37.80	3.38–9.08	3.79–32.75	102.18–135.40	135.40–155.69	137.85–149.28	36.14–68.86	87.24–105.35	89.66–98.34	67.88–92.41		
**Traits**	week 1		*Under*	Bipolar								1.35	1.87
	week 2		Under	Bipolar								0.81	0.98
	week 3		Paternal	Bipolar								0.87	1.11
	week 4			*Bipolar			*Paternal*				Paternal	1.85	3.03
	week 5			*Bipolar	Bipolar	Paternal	**Paternal**	**Paternal**		**Over**	**Paternal**	3.35	10.38
	week 6	**Paternal**		Bipolar	*Bipolar*	*Paternal*	Paternal	**Paternal**		**Over**	**Paternal**	4.58	13.19
	week 7	**Paternal**		Maternal	*Bipolar*	*Paternal*	Over	**Paternal**	Over	*Over*		5.67	15.77
	week 8	**Paternal**			*Bipolar*	*Paternal*	Over	**Paternal**	Over			4.63	11.95
	week 9	**Paternal**		Maternal	*Bipolar*	Paternal	Over	**Paternal**	Over			5.54	12.29
	week 10			Maternal	*Bipolar*	*Paternal*	Over		Over			4.84	9.92
	growth 1–3							*Paternal*				0.80	1.72
	growth 3–10	**Paternal**			*Bipolar*	Paternal	Over	**Paternal**	*Bipolar*			5.34	10.53

The locations are given in F_2_ cM and the confidence intervals are based on the genome coordinates of the markers that flank the confidence region (see Methods). Coordinates (Mb) are based on mouse genome build 36 (www.ensembl.org). The best fit pattern of a locus for each trait is listed as Paternal = paternal expression, Maternal = maternal expression, Bipolar = bipolar dominance imprinting, Over = polar overdominance imprinting and Under = polar underdominance imprinting. Patterns where the *i*QTL was significant at the genome-wide level are shown in bold, patterns significant at the chromosome level are italicized and patterns significant at the locus level are shown in plain text (see Methods). Patterns marked with an asterisk show a parent-of-origin effect (*i*) that is significant at the chromosome level, but the overall model test for the locus is not significant. These are included as suggestive *i*QTL because of the strength of their putative imprinting effect. Shown are the 10 weekly weights and two growth traits (growth 1–3 is weight gain from week 1 to week 3 while growth 3–10 is weight gain from week 3 to 10). The dashed line indicates the age of weaning (included to highlight that nearly all effects occur post-weaning). Values of effects and exact significance values are given in Table S1.

Genomic imprinting was found to affect all weekly weights and growth traits. The effects of the *i*QTL are generally pleiotropic, with effects on weight at different stages in development. The effects of four of these loci show a change in the imprinting pattern over time (Table 1). The dashed line in Table 1 indicates the weaning age, and it is noteworthy that almost all *i*QTL effects occurred after weaning. Imprinted QTL for weight and growth are identified as *WtiX.Y* where *Wt* specifies body weight, *i* specifies an imprinting effect, *X* specifies the chromosome and *Y* specifies the *i*QTL on the chromosome that is being referred to. On chromosome 3 we detected several *i*QTL, with the proximal QTL (*Wti3.1*) being more than 50cM away from the distal (*Wti3.2*) and the two are thus regarded as independent from each other. In addition, two-QTL mapping analysis (see Methods) revealed a third QTL on distal chromosome 3 (*Wti3.3*).

**Figure 1:**
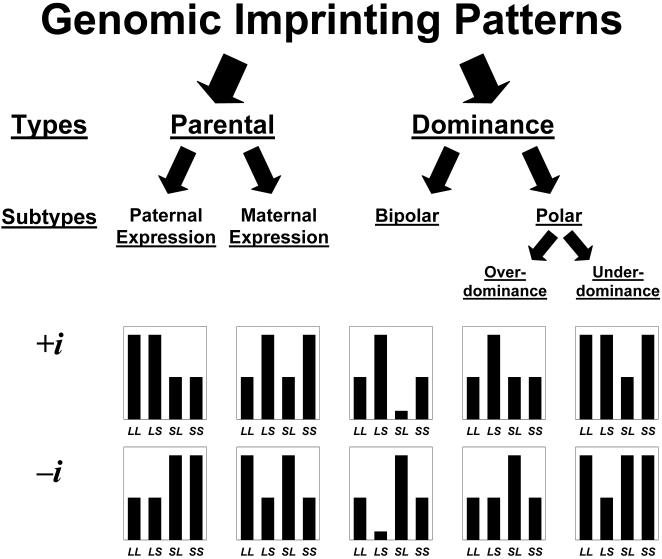
All possible phenotypic patterns of genomic imprinting. Two principal patterns are possible: parental expression and dominance imprinting. Parental expression has two subtypes describing which allele is being expressed (paternal versus maternal). Dominance imprinting refers to the case where the two homozygotes are the same while the heterozygotes are different from each other. There are two subtypes of dominance imprinting: bipolar and polar. Bipolar dominance refers to the case where one heterozygote is larger than the homozygotes while the other heterozygote is smaller (i.e., one heterozygote shows overdominance while the other shows underdominance). Polar dominance refers to the case where one heterozygote is the same as the two homozygotes while the other heterozygote is not. Polar dominance may show overdominance, where the heterozygote differing from the other three genotypes is larger, or underdominance, where it is smaller. The plots give examples of the expected pattern of phenotypes for the four ordered genotypes when the sign of *i* is either positive or negative.

**Figure 2:**
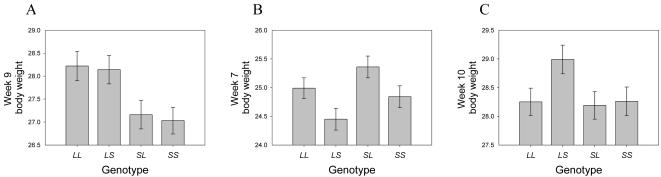
Three examples of imprinting patterns found in the genome-wide scan. Each of the four genotypes is shown with its corresponding average phenotype plus standard error of the mean at the locus. A) *Wti1.1* serves as an example for paternal expression for week 9 body weight (g). B) *Wti3.2* provides an example of bipolar dominance for week 7 body weight (g), C) *Wti5.1* provides an example of polar overdominance for week 10 body weight (g).

**Figure 3:**
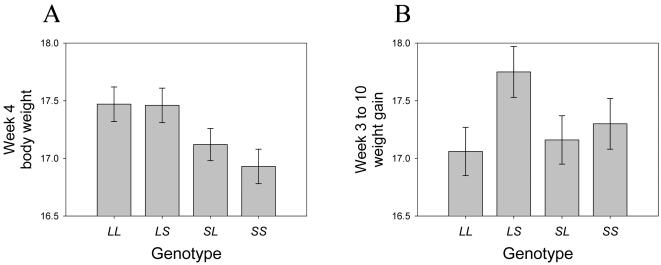
Change in imprinting pattern for *i*QTL *Wti5.1* caused by imprinting effects on growth. For week 4 body weight (g) the locus shows paternal expression (A), but later the locus shows polar overdominance (see Figure 2A for the pattern in week 10). The change in the pattern of imprinting is due to polar overdominance for growth from week 3 to 10 (B), where the *LS* heterozygote grows more than the other three ordered genotypes. Error bars denote standard errors of the mean.

Across all loci, we discovered five different patterns of imprinting: paternal expression, maternal expression, polar over- and underdominance imprinting and bipolar dominance. Figure 1 and Material and Methods contain a description of the forms of imprinting, which are defined by the relationship of the imprinting genotypic value *i* to the additive and dominance genotypic values (*a* and *d*) as well as their sign. Six loci showed paternal expression at some point during development (e.g. *Wti1.1*; Figure 2A), with four of these showing exclusively paternal expression through development. Only one locus (*Wti3.1*) showed maternal expression. Three loci showed a hitherto undescribed pattern which we refer to as bipolar dominance (e.g. *Wti3.2*; Figure 2B) where the two heterozygotes are significantly different from each other but the two homozygotes have similar phenotypes and are not different from each other. Four loci showed polar dominance imprinting (e.g. *Wti5.1*; Figure 2C), with three of the four showing polar overdominance. Most loci maintained the same pattern over ontogeny, however, four loci showed a change in expression pattern through time. For example, *Wti5.1* showed paternal expression in week 4 (Figure 3A), but the pattern gradually changed to polar overdominance through time. By week 7, the best fit model was for polar overdominance, and by week 10 the pattern was very clearly polar overdominance (Figure 2C). The change in the pattern of the ordered genotypes was caused by polar overdominance for growth after weaning (Figure 3B), with the *LS* heterozygote growing faster than the other three genotypes. Similarly, locus *Wti2.1* showed bipolar dominance early in development (with the bipolar pattern being the best fit pattern from week 1 to week 6), but by week 7 the pattern had shifted to maternal expression. Likewise, *Wit2.1* changed from polar underdominance to paternal expression from week 1 to week 3.

**Figure 4:**
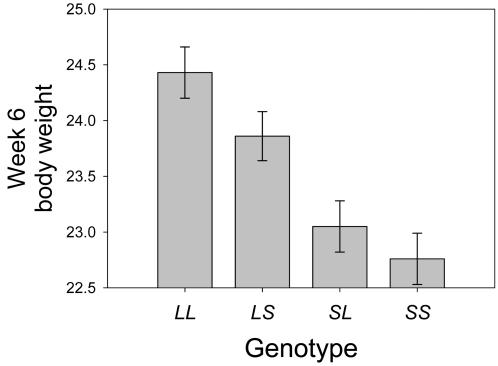
A pattern of paternal expression associated with partial imprinting. Locus *Wti7.1* shows a pattern of paternal expression for week 6 body weight (g) where the difference between the genotypic values of the heterozygotes (0.81g) is less than half the difference between the homozygotes (1.66g).

Many of the loci showed patterns consistent with partial imprinting, where the difference between the two homozygotes is larger than the difference between the two heterozygotes. This can be seen in the relationships between the additive or dominance genotypic values to the imprinting genotypic value (*a*/*i* or *d*/*i*; see Table S1), which deviate from the values expected for a particular form of imprinting. For example, most loci showing paternal expression have much larger additive effects than imprinting effects; resulting in *a*/*i* ratios larger than 1. In many cases of paternal expression, the additive effect is more than twice the imprinting effect; this is illustrated in Figure 4 for the effect of locus *Wti7.1* on week 6 weight, where the additive effect is just over twice the imprinting effect.

The effects of the *i*QTL (including their additive, dominance and imprinting effects) together accounted for between 1 and 15.8% of the phenotypic variance in age-specific weights and weight gain, with the imprinting effects alone accounting for between 0.8 and 5.7% of the phenotypic variance. The overall effects of individual loci (i.e., their additive, dominance and imprinting effects together) explained between a 0.34 and 6.5% of the phenotypic variance (with an average of 1.6%), with the imprinting effects alone accounting for between 0.2 and 1.7% of the phenotypic variance. Surprisingly, the strongest effect of genomic imprinting on weight occurred between weeks 6 and 10 (Table 1).

Turning to the duration of imprinting effects and their onset we found that most imprinted loci showed significant effects over long periods during development, with only a single locus (*Wti2.1*) showing effects limited to the pre-weaning period. The QTL with imprinting effects over the longest period is located on proximal chromosome 3 (*Wti3.1*) with a significant effect on most weights from week 1–9.

## Discussion

This study advances research on genomic imprinting in several ways. First, by using genotypes from two rather than one generation we can assign parent-of-origin of alleles with near certainty without invoking probabilities to calculate likelihoods of allelic parent-of-origin. Thus, we have been able to examine in detail the pattern of phenotypic variation caused by genomic imprinting, and found previously unknown patterns of imprinting. Second, these results suggest that imprinting patterns may be more diverse and, consequently, the traditional view of predominantly paternally or maternally expressed loci should be replaced with a picture of multiple imprinting patterns (Figure 1). Indeed, most *i*QTL detected in our study display patterns other than simple paternal or maternal expression, with three loci showing the new bipolar dominance imprinting pattern. Third, an important implication of our results is that the effects of alleles may change sign depending on their parent-of-origin (see below). These parent-of-origin-dependent allelic effects may also be akin to dominance in that the effect of an imprinted allele not only depends on its parent-of-origin, but also on the allele it is paired with at a locus. Finally, the results of this study demonstrate that imprinting effects can vary over time both in their patterns (Figure 3) and the proportion of variance explained, and may arise or persist well into adulthood. The latter highlights that imprinting effects are not necessarily most influential at early stages in development as currently viewed [e.g. 16].

The processes underlying the diversity of imprinting patterns found in our study are likely due to different mechanisms [23] many of which may involve differentially methylated DNA elements called imprinting centres regulating multiple genes in a region [24]. Wood & Oakey [23] discuss three different mechanisms that may explain uniparental expression patterns. While the enhancer-blocker model invokes an imprinting centre between reciprocally expressed genes with shared enhancer elements (e.g. *Igf2/H19*), a second model for the maternally expressed *Igf2r* gene utilizes *cis*-mediated silencing of maternally expressed genes by non-coding paternally expressed RNA (e.g. *Igf2r*). Finally, at microimprinted domains oocyte-derived methylation in the promoter region of protein-coding genes is assumed to be the key mechanism. In addition to the ‘traditional’ imprinting patterns we found four loci with polar dominance effects causing a pattern equivalent to that described for the callipyge (*CLPG*) locus in sheep and pig homologues *DLK1-GTL2* [17],[25] where one of the two heterozygotes is different from the other three genotypes [26]. For the callipyge locus, the observed pattern is caused by a paternally inherited mutation in the *CLPG* locus that results in the expression of a number of core group genes in *cis* in addition to an interaction in *trans* between reciprocally imprinted genes [26]. The authors proposed that inhibition of DNA methylation or altered histone modification may be causal to the callipyge phenotype. To our knowledge, a pattern of polar dominance has only once been reported previously for any known murine gene [15], and in that case the locus effect was lethality, not trait expression. Furthermore, while polar overdominance has been found for one locus in sheep and pigs, no prior studies in any system have observed a pattern of polar underdominance imprinting affecting trait expression as demonstrated by our results.

**Figure 5:**
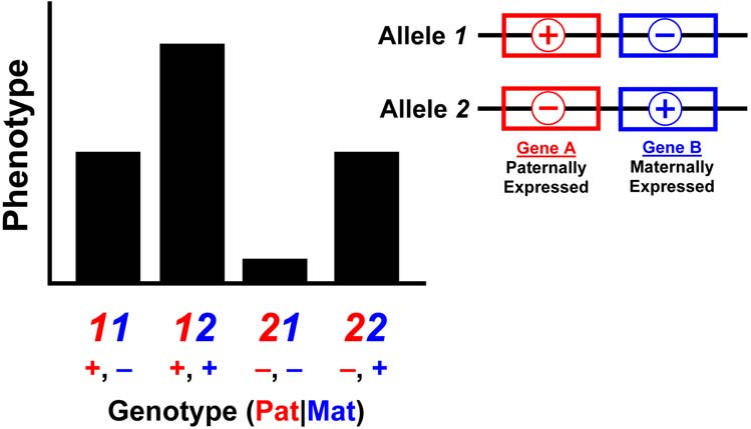
A hypothetical model explaining the appearance of bipolar dominance imprinting. In this model, a QTL with two alleles (*1* and *2*) is comprised of two genes (*A* and *B*), which are in close linkage. Gene *A* is paternally expressed while *B* is maternally expressed. Allele *1* has a positive effect on a trait when paternally inherited but a negative effect when maternally inherited while allele *2* shows the opposite pattern. When the same allele is inherited from both the father and the mother the effects cancel out, yielding no difference between the two homozygotes. However, if two different alleles are inherited from the parents then the joint effects of the paternal and maternal copy do not cancel anymore but produce a pattern of bipolar dominance.

We suggest that the pattern of bipolar dominance may be explained by a model where the sign of the allelic effect changes depending on the parent-of-origin. This might occur when two differentially imprinted genes are in close linkage (e.g. callipyge), such that the alternative alleles are composed of variants at both the maternally and the paternally expressed loci. This scenario is illustrated in Figure 5, showing a hypothetical case in which a QTL with alleles *1* and *2* is comprised of two variable sites, *A* and *B*, that are in close linkage, with gene *A* being paternally expressed while *B* is maternally expressed. The effect of the paternally derived QTL copy will be determined by variation at site *A* while the effect of the maternally inherited copy will be determined by variation at site *B*. In this scenario, allele *1* of the QTL may have a positive effect on a trait when paternally inherited but a negative effect when maternally inherited whereas allele *2* may show the opposite pattern. When the same allele (*1* or *2*) is inherited from both the father and the mother the effects cancel out, yielding no difference between the two homozygotes. However, if two different alleles are inherited from the parents then the joint effects of the paternal and maternal copy do not cancel and, as a result, produce a pattern of bipolar dominance (Figure 1). As with the callipyge locus in sheep [26], this pattern of ‘interference’ between closely linked maternally and paternally expressed loci could potentially be a signature of conflict, where concerted counter-evolution of maternally and paternally expressed alleles results in linked alleles that negate each other's effect.

The currently known number of imprinted loci in mice (about 80; www.geneimprint.com) may in part reflect a research bias toward regions of the genome with chromosomal aberrations and loci with large phenotypic effects, especially in the light of recent research showing that as many as 600 genes are predicted to be imprinted [19]. First, comparing the locations of *i*QTL found in our study with those of currently known imprinted genes (www.geneimprint.com), we find that most of our loci are likely to be novel. No currently known imprinted genes are located on chromosomes 1, or 3, where we detected a total of four *i*QTL. There are known imprinted genes on chromosomes 2 and 5, but they all lie well outside of the confidence intervals [27] for the *i*QTL locations (ca. 100Mb away on chr. 2, 40Mb away on chr. 5). Chromosome 12 has a number of imprinted genes that are located close to but outside of either end of the confidence interval, with *Mirn337* more than 20Mb proximal and several genes (*Dlk1m*, *Gtl2*, *Rtl1*, *Dio3*) more than 10Mb distal to the confidence interval. The *i*QTL with the strongest effect (*Wti7.1*) is located on Chromosome 7, which contains nearly half of the currently confirmed imprinted genes in mice (www.geneimprint.com). The confidence interval for the location of this locus includes 17 known imprinted genes (ca. 20–24% of all currently confirmed or putatively imprinted genes in mice; www.geneimprint.com), including for example, *Peg3*, *Peg4* (*Snrpn*), *Peg6* (*Ndn*) and *Peg12*. More than half of these (10 of 17) and 10 of 14 loci with a known imprinting pattern are characterized by paternal expression, which matches the pattern we identified for the *i*QTL in this region. On chromosome 9, the imprinted gene *Rasgrf1* falls within the confidence region of *Wti9.1. Rasgrf1* was the first imprinted gene found to affect postnatal growth only [5] and has been described as paternally expressed. While the postnatal effect of *Rasgrf1* is congruent with the effect on postweaning growth found for *Wti*9.1, we found a bipolar pattern for growth and polar overdominance for weekly weights affected by this locus in contrast to paternal expression reported for *Rasgrf1*. However, we note it is unclear whether a bipolar pattern would emerge for *Rasgrf1* for the post-weaning growth period of 3–10 weeks. Further studies both on the growth trajectories of *Rasgrf1* mutants and on fine-mapping our identified *i*QTL are required to determine whether *Rasgrf1* could be a candidate gene. Finally, the confidence interval for the location of *Wti14.1* includes a single known imprinted gene, *Htr2a*, which is known to be maternally expressed in mice, in contrast to the pattern of paternal expression seen for the *i*QTL, suggesting it is an unlikely candidate gene.

Turning to the results of Luedi et al. [19] whose simulation study predicted 600 imprinted genes across the genome, we found that a total of 50 predicted genes are within the confidence regions of our *i*QTL (Table 2). While one may expect some congruence of our confidence regions and the list of predicted genes from Luedi et al. by chance (5 per QTL locus), several confidence regions contain a large number of predicted imprinted genes (and the predicted imprinted genes are not uniformly distributed across the genome). Confidence intervals for all loci contain multiple genes predicted to be imprinted, providing potential candidate genes for all *i*QTL that could be explored in future fine mapping and methylation-status studies to search for imprinted genes.

**Table 2:** Predicted imprinted genes (Luedi et al. 2005) lying within the confidence regions of our *i*QTL.

Ensembl ID	Gene	Chr.	Expr.	Ensembl ID	Gene	Chr.	Expr.
***Wit1.1***				***Wti5.1***			
ENSMUSG00000026158	Q8VE52	1a5	P	ENSMUSG00000047105		5g1	M
ENSMUSG00000033569	Bai3	1a5	M	ENSMUSG00000041132	AI428195	5g3	M
ENSMUSG00000041670		1a5	M				
ENSMUSG00000026110		1b	M	***Wti7.1***			
				ENSMUSG00000051425	1810013P09Rik	7b1	M
***Wti2.1***				ENSMUSG00000039257	AB030198	7b2	P
ENSMUSG00000037228		2a1	M	ENSMUSG00000025324	Atp10a	7b5	M
ENSMUSG00000051576		2a1	P	ENSMUSG00000047469		7c	M
******				******			
***Wti3.1***				***Wti9.1***			
ENSMUSG00000040289	Hey1	3a1	P	ENSMUSG00000032423	Nsap1-pending	9e3.2	P
ENSMUSG00000049478		3a2	M	ENSMUSG00000032353	1200002G13Rik	9e3.2	M
ENSMUSG00000049569		3a3	P	ENSMUSG00000032422	NM 172926	9e3.2	P
ENSMUSG00000027630		3a3	P	ENSMUSG00000032456	4933408N02Rik	9e4	P
ENSMUSG00000002428	Smarca3	3a3	M	ENSMUSG00000047985		9f1	M
				ENSMUSG00000023495	Pcbp4	9f1	M
***Wti3.2***				ENSMUSG00000032470	Mras	9f1	M
ENSMUSG00000027859	Ngfb	3f3	P				
ENSMUSG00000000001	Gnai3	3f3	M	***Wti12.1***			
ENSMUSG00000033161	Atp1a1	3f3	M	ENSMUSG00000034389		12e	M
ENSMUSG00000050461		3f3	P	ENSMUSG00000021209	8430415E04Rik	12f1	P
ENSMUSG00000051638		3f3	P	ENSMUSG00000033879		12f1	M
ENSMUSG00000044869		3g1	P	ENSMUSG00000044456		12f1	M
ENSMUSG00000027987		3h1	M				
ENSMUSG00000037994		3h2	P	***Wti14.1***			
ENSMUSG00000046818	NM 030143	3h2	M				
				ENSMUSG00000015807	NM 172811	14d1	P
***Wti3.3***				ENSMUSG00000051398		14d1	P
ENSMUSG00000037994				ENSMUSG00000021998	Lcp1	14d2	P
ENSMUSG00000046818	NM 030143	3h2	P	ENSMUSG00000022002	4930564B18Rik	14d2	P
ENSMUSG00000028194	Ddah1	3h2	M	ENSMUSG00000042930		14d2	P
ENSMUSG00000028195	Cyr61	3h3	M	ENSMUSG00000022019	NM 172605	14d3	M
ENSMUSG00000036745	4921517B04Rik	3h3	M	ENSMUSG00000022021	Diap3	14d3	M
ENSMUSG00000028036	Ptgfr	3h3	P				
ENSMUSG00000028199	Cryz	3h4	P				
ENSMUSG00000040037	NM 177274	3h4	P				

The column headed ‘Chr.’ lists the chromosomal bands based on mouse genome build 36. The predicted expression pattern is denoted M = maternal expression and P = paternal expression.

Somewhat surprisingly, we found only two loci that show imprinting effects during the pre-weaning period from 1 to 3 weeks and only one of these loci (*Wti2.1*) has effects that are restricted entirely to the preweaning period. Many more loci show effects that do not appear until week 5 or later and many of these extend to mature adult weight at 9 or 10 weeks. Additive and dominance QTL mapping in this population of mice has shown that different sets of QTL affect variation in growth and body weight between 1 and 3 weeks of age and between 4 and 10 weeks [28],[29], paralleling the known differences in the physiology of mammalian growth over these periods [30]. These previous analyses of additive and dominance effects have shown that the number of QTL affecting weekly weights and growth does not vary greatly before and after weaning, but that dominance effects tend to be more important earlier (peaking around weaning) while additive effects tend to increase in magnitude with age. Following these results we have no reason to assume that a bias exists for finding imprinting effects before or after weaning.

Overall, our results suggest that the quantitative analysis of imprinting effects using allelic variation can identify genomic regions showing novel imprinting effect patterns (e.g., bipolar dominance). Moreover, by not restricting our analysis to traits expressed early in life we demonstrate that imprinting effects can appear and often be stronger later in life (and notably, after the cessation of maternal care), and may also change their pattern of effect during growth and development. More generally, our investigation provides a framework for classifying the diversity of patterns that imprinted loci may show (Figure 1). Further investigation into the proximate causes of the underlying processes that generate these novel imprinting patterns may ultimately provide important insights into the evolutionary origin of imprinting and multiple pathways in which imprinting contributes to quantitative trait variation.

## Material and Methods

### Animal Husbandry and Phenotypes

We used the F_2_ and F_3_ generation of an intercross between the inbred mouse strains Large (LG/J) and Small (SM/J) [31]. These strains were established over 60 years ago and were originally under artificial selection for either large or small body weight at 60 days of age [21],[32],[33] and have been inbred for over 120 generations prior to their use in this study. Due to this extended period of inbreeding, these strains are essentially devoid of within-strain variation. The strains differ by 6–8 standard deviations in size and growth related traits [31], making this an ideal model system to study imprinting effects arising from genes regulating growth and development. To generate the study population, ten males of the SM/J strain were mated to ten females of the LG/J strain. The resulting F_1_ population consisted of 52 individuals, which were randomly mated to produce 510 F_2_ animals, representing the parental generation in our study. These F_2_ animals, again, were randomly mated to produce 200 full-sibling families of the F_3_ generation with a total of 1632 individuals. Males were removed from the cages when females were visibly pregnant. Half litters were reciprocally cross-fostered at random between pairs of females that gave birth on the same day. In total, offspring in 158 families were cross-fostered in this way. Pups were weaned at 21 days of age and randomly housed with three or four other same sex individuals. Further details of the husbandry are given in [28],[29].

Pups were weighed weekly starting at one week of age through week 10 using a digital scale with an accuracy of 0.1g. Growth was calculated as the difference between weekly weights such that, for example, the growth from week 1 to week 3 is the difference between week 3 weight and week 1 weight. The traits analysed in this study are weekly individual bodyweights corrected for sex and litter size beginning with weight at week 1 and ending with weight at week 10. Growth traits were obtained for preweaning growth from week 1 to 3 and for the postweaning growth from week 3 to 10.

### Genotyping

DNA was extracted from livers of the F_2_ and F_3_ individuals using Qiagen DNeasy tissue kits. After standardizing DNA concentration, the samples were scored for 384 SNPs using the Golden Gate Assay by Illumina, San Diego, USA. These markers were previously found to be polymorphic between LG/J and SM/J as part of the Oxford/CTC genotyping collaboration (http://www.well.ox.ac.uk/mouse/INBREDS/). After further testing, 15 loci were found not to have been reliably scored and were excluded from the analysis. Sixteen loci were scored on the X chromosome and are not included in this analysis because the genome structure and the statistical model for the X are complex and unresolved. This leaves 353 loci across the 19 autosomes for analyses. A genetic map of these markers based on Haldane's centiMorgans (cM) was produced using R/QTL [34] and validated against the genome coordinate locations in the Ensembl database (www.ensembl.org). The average map distance between markers in the F_2_ generation is 4 cM. Markers are evenly placed throughout the genome except for regions in which LG/J and SM/J have been found to be monomorphic [35]. A list of the markers along with their physical and map positions are given in Table S2.

### Haplotype Reconstruction

The combined genotypes of parents and offspring were used to reconstruct haplotypes for all animals with the program PedPhase [36],[37], which uses several algorithms to infer haplotype configurations for all individuals that minimize the number of recombination events in the whole pedigree [i.e., it solves the ‘minimum-recombination haplotypes configuration problem’; 38]. We used the ‘block-extension algorithm’ to reconstruct haplotypes, which produced a set of unordered haplotypes for the F_2_ animals and a set of ordered haplotypes (i.e., ordered by parent-of-origin of alleles) for the F_3_ animals. The ordered genotypes of the F_3_ allowed us to distinguish between the four possible genotypes at a given locus, *LL, SL, LS* or *SS* (*L* being the LG/J allele and *S* the SM/J allele) where the first allele refers to the paternally derived allele and the second to the maternally derived allele.

### Analysis of Parent-of-Origin-Dependent Effects

The four ordered genotypes at the marker loci (*LL, LS, SL* and *SS*) were assigned additive, dominance and imprinting (parent-of-origin) genotypic index values following Mantey et al. [15]. These index values (slightly modified from those used by Mantey et al. ref. 15) can be written in matrix form, where the vectors of genotypic means (i.e., genotypic values) (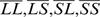) are defined by:
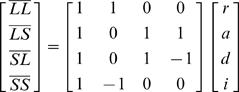
(1)which yields estimates of the parameters:
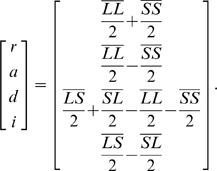
(2)Where *r* is the reference point for the model (the mid-point between homozygotes), *a* is the additive genotypic value (half the difference between homozygotes), *d* is the dominance genotypic value (the difference between the mean of the heterozygotes and the mid-point of the homozygote means), and *i* is the parent-of-origin or imprinting genotypic value (half the difference between heterozygotes) [cf. ref. 15].

These index values in equation (1) were used to build a model to scan the genome in the F_3_ generation (parent-of-origin of alleles cannot be directly assigned in the F_2_ because their F_1_ parents are all genetically identical, making it impossible to unambiguously assign haplotypes to parents) to detect loci showing significant parent-of-origin-dependent effects (i.e. significant *i* effects). A mixed general linear model was used to estimate the overall significance of a locus as well as the significance of the additive, dominance, and imprinting effects. To test the overall significance of a locus, a model with ordered genotype class as a fixed effect and family as a random effect was fitted using restricted maximum likelihood (REML) as implemented in the Mixed Procedure of SAS (SAS version 9.1; SAS Institute, Cary, NC, USA). The significance of the individual genetic effects was determined using a mixed model with the *a*, *d* and *i* index values as fixed regression effects and family as a random effect (fitted again using REML in the Mixed Procedure of SAS). Family was included as a random effect to account for variation among families not attributable to the effects of the locus in question. A power analysis combined with a simulation to determine significance thresholds (see below) showed that the inclusion of family greatly improved power while also removing any bias in significance tests introduced by family structure.

The mixed model with the fixed genetic effects and random family effect was used to scan the genome to produce a probability distribution for the overall effect of the locus as well as the additive (*a*), dominance (*d*) and imprinting (*i*) effects. These probability values were then transformed to a logarithmic probability ratio (LPR) in order to make them comparable to the LOD scores typically seen in QTL analyses (LPR = −log_10_(probability)). Significance thresholds were determined using a Bonferroni correction, which was calculated using the effective number of markers method [39], which has been demonstrated to be less artificially conservative than a simple Bonferroni correction. This analysis showed that, due to correlations between linked markers, the genome has 133 effective markers, which results in a Bonferroni threshold LPR at the 5% level (i.e., *α* = 0.05) of 3.41. Chen & Storey [40] have shown that, where several QTL can be expected to affect traits, a modified genome-wide error rate should be applied as opposed to the traditional genome-wide error rate or the false discovery rate. This is achieved by applying the significance criterion to the highest LPR on each chromosome and yields overall the best results by increasing the discovery of true positives while at the same time avoiding problems using the false discovery rate in gene mapping experiments (with 19 autosomes, we would expect only about 1 false positive result using the chromosome level thresholds). Our collective chromosome-wide significant results across the genome greatly surpass this expectation providing confidence in the overall set of results. Therefore, for each chromosome we used the effective number of markers on the chromosome [40] to generate a chromosome-level significance threshold. The thresholds for individual chromosomes are given in the Supplementary Table. Once a QTL was identified, we used post-hoc tests, with a LPR significance threshold of 1.3 (i.e., *p*<0.05) to determine whether the locus also had additive, dominance or imprinting effects, or affected more than one trait. Confidence intervals for the positions of *i*QTL were determined using a one LOD drop (using LPR values) following Lander & Botstein [27].

Because apparent parent-of-origin effects at a locus can also be caused by a maternal effect of that locus, rather than genomic imprinting, we tested all loci with a significant *i* effect to confirm that the appearance of a parent-of-origin effect could not be attributed to maternal effects [22]. If the apparent imprinting effect is due to a genetic maternal effect, the differences between reciprocal heterozygotes born of homozygous mothers will be much larger than the differences between those born of heterozygous mothers, which are all exposed to the same maternal environment. Therefore, we confirmed the existence of imprinting by testing the *i* effect using only the offspring from heterozygous mothers. This approach adequately accounts for the potential confounding patterns of maternal effects since maternal effects only lead to the appearance of a parent-of-origin dependent effect at the locus that has the maternal effect. The occurrence of non-genetic maternal effects cannot lead to the appearance of parent-of-origin dependent effects and, likewise, the presence of maternal genetic effects attributable to other loci in the genome will not lead to the appearance of a parent-of-origin effect at other loci.

To determine the relative proportion of variance explained by the loci overall and by genomic imprinting effects, we calculated the approximate variance contributed by a locus (*Vg*) using the expectation:
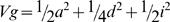
(3)which is the genetic variance of a locus in a population with two alleles at approximately equal frequency in Hardy-Weinberg equilibrium. The analytical expectation was used because REML does not compute sums of squares and the corresponding *R^2^*. The proportion of variance explained would, therefore, be *Vg*/*Vp* (*Vp* being the phenotypic variance). To obtain the variance explained by the parent-of-origin effect alone we calculated 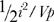


When chromosomes contained more than one significant QTL, we assumed that all loci more than 50cM apart represented separate *i*QTL. For cases where loci were closer than 50cM apart, we tested a model containing the individual loci to confirm that both were significant in a combined model.

### Analysis of Imprinting Patterns

We characterized the patterns of imprinting at QTL by comparing the relative fit of different possible imprinting patterns. Generally, it is assumed that imprinting leads to monoallelic expression [e.g. 10], where only one allele is expressed and the other is always silent (i.e. ‘maternal’ or ‘paternal’ expression). Alternatively, there could be partial silencing of one allele, with both alleles being expressed but to different degrees [18],[41]. In addition to these patterns based on complete or partial silencing of alleles, significant imprinting could appear as a result of a number of other more complex patterns (below), which to our knowledge have not been investigated to date.

To characterize patterns of imprinting, we examined the relationships between the three genotypic values (*a*, *d* and *i*; see Table S1) to define a set of basic patterns of imprinting as illustrated in Figure 1. The patterns shown in Figure 1 are idealized and are used as a general classification system. Actual patterns may differ from these due to the fact that loci may show tissue specific variation in imprinting patterns, or because of partial imprinting. We determined the best-fit imprinting pattern at a given locus using contrasts in the Mixed Procedure in SAS. The various imprinting patterns are reflected in distinct ratios of the genotypic value for imprinting *i* and the additive *a* and dominance *d* genotypic values and yield the following different imprinting patterns.

1. With complete or partial monoallelic expression (corresponding to either maternal or paternal expression), we expect that the two genotypes sharing the same expressed allele should have the same average phenotype. Complete silencing of one allele implies that (*a/i*) = *+*1 if there is paternal expression and (*a/i*) = *−*1 for maternal expression, and also that *d/i* = 0. A locus was said to show partial maternal or paternal expression when the (*a/i*) ratio was closer to zero but both *a* and *i* were still statistically significant or when the ratio was much larger than one. To detect maternal expression, the *LL* and *SL* genotypic values were contrasted with the *LS* and *SS* genotypic values (i.e., genotypes sharing alleles with the same maternal origin), while the opposite contrast was used to detect paternal expression.
2. A locus with a significant *i* effect may also show a previously undescribed imprinting pattern, where the two heterozygotes are significantly different from each other, but the two homozygotes are the same. We call this pattern of imprinting ‘bipolar dominance’ because one heterozygote shows overdominance while the other shows underdominance. Bipolar dominance is characterized in its canonical form by a significant *i* value with additive (*a*) and dominance (*d*) values of zero, thus *d/i = *0 and *a/i = *0. This pattern cannot be attributed to simple silencing since such a process would necessarily result in a difference between the homozygotes. Possible mechanisms producing this pattern are discussed in the main text. To detect bipolar dominance, the *SL* genotypic value was contrasted with the *LS* genotypic value.
3. A third possibility is a more general case of the pattern previously called polar overdominance [17]. This pattern of imprinting, which we call ‘polar dominance’, refers to the situation when one of the two heterozygotes is different from all three other genotypes (either significantly larger or smaller) while the genotypic values of the latter are not significantly different from each other. The pattern is referred to as polar overdominance when the heterozygote is larger than the other three ordered genotypes and polar underdominance when it is smaller. The case of polar overdominance matches that described for the *callipyge* locus in sheep [17]. In its canonical form, this pattern is associated with a dominance value (*d*) that is equal to the imprinting value (*i*) and with an additive effect (*a*) of zero, thus *d/i = ±*1 and *a/i* = 0. To detect polar dominance, either the *LS* or *SL* genotypic values were contrasted with the values of the other three genotypes.

We note that actual genotypic values may deviate from the definitions given above and patterns with genotypic value ratios approximating −0.5 or 0.5 cannot be unequivocally categorized.

### Simulation and Power Analysis

We simulated the production of the F_2_ and F_3_ populations maintaining the observed distribution of family size to evaluate power of alternative mapping approaches (specifically, including random family effects and testing model significance based on the significance of the full effect of a locus (*a*, *d*, and *i*) versus just the imprinting effect (*i*) and the possibility of inflation of significance thresholds caused by family structure). F_2_ animals' genotypes were produced by combining recombinant gametes from their F_1_ parents. We used the recombination rates observed in the F_2_ population. F_2_ animals were randomly paired and gametes produced for each offspring, again using the same recombination rates and the observed distribution of family sizes. A thousand random independent loci, one from each of a thousand iterations of the simulation, were used for power analyses. These family-structured genotypes were then paired with the observed phenotypes. Thus, each simulated locus had the same distribution of genetic correlation between individuals as the actual population. The distribution of phenotypes within and between families was also maintained, fixing heritability and genetic correlation. However, in the simulated population there is no relationship between any specific locus and the distribution of phenotypes, unless such a relationship was simulated.

QTL were simulated by altering the phenotypes of individuals based on their genotype at a locus in a way that simulated two patterns of imprinting, parental imprinting and bipolar dominance, and two patterns of QTL effects that did not include imprinting, pure additivity and overdominance effects. QTL effects were simulated following the definition of the genotypic values given in equation (1) in a way that did not alter the trait mean. QTL were simulated to account for ½, 1, 2 and 5% of the phenotypic variance according to the relationship shown in eq. (3) for *Vg* (where the phenotypic variance is calculated as the existing variance plus the variance contributed by the simulated QTL, such that the QTL effects are measured relative to the final phenotypic variance after the addition of variation contributed by the QTL).

Because our goal is to present an analysis of imprinting effects and not a general analysis of statistical approaches, we only briefly present the relevant details and results here in the Materials & Methods section. We use results of a simulation of week 10 body weight because this trait has a heritability that is approximately equal to the median and modal heritability of the 12 traits analyzed herein. We fitted the model (both with and without the random family effect) using REML as described above and focus on whether the inclusion of the family effect improves power and how often the contrasts that test the fit of the locus to the various forms of imprinting correctly identify the true pattern. We also evaluated the case where significance testing is based solely on the imprinting effect to see whether this improves the detection and correct characterization of imprinting effects.

The null model with no QTL effects shows that the threshold for a model not including the random effect of family (i.e., a model with just the fixed family effect) has an inflated significance threshold due to the presence of the family structure (i.e., the familial autocorrelation), where the 5% significance threshold is 0.0007, rather than 0.05 as expected. In contrast, the model that includes the random effect of family in the mixed model has a 5% significance threshold of 0.048. This shows that the inclusion of the random effect of family, by removing the among family variance, removes any bias in significance tests caused by family structure. Because we have identified loci using a test of the overall effect of a locus prior to determining whether the locus shows imprinting, our expected rate of false positives is actually much lower than the 5% expected based on a 5% significance threshold for the locus. This is because most loci showing a significant overall effect do not show an imprinting effect. Indeed, when the fixed effect of family is included in the model, we find that, as expected, only a fraction of the false positives show imprinting, indicating that the actual rate of false positives for loci showing an imprinting effect is just over 1%. When the model does not include the random family effect, we find that the false positive rate of loci showing imprinting is just over 2%.

Table S3 presents the results of the simulations that included imprinting effects. In all cases, the model that included the random family effect performed better than the model that did not. In all cases, the inclusion of the family effect increased power to detect QTL effects (generally a two- to threefold increase) compared to a model without family and always yielded a higher proportion of correct assignment of the pattern of QTL effects. Therefore, we have included the random family effect in the model and all further discussion of power is based on the model that includes the family effect. Overall power of the mixed model fitted using REML is high and generally identifies the correct pattern of imprinted expression. The power analysis shows that, when the locus shows parental imprinting, the use of a significance test based on the overall effect of the locus has higher power and is correct a greater proportion of the time compared to the use of the significance test based on the imprinting effect alone. However, when the locus shows bipolar dominance (especially when it is weak, accounting for ½ or 1% of *Vp*), power is higher when the test is based solely on the imprinting effect, but the proportion showing the correct pattern is generally similar to the test based on the overall effect of the locus. The increased power is due to the fact that the bipolar pattern only contributes to the imprinting term, and therefore, a test based on the overall effect of the locus is less sensitive than one based solely on the imprinting effect. For this reason, we have included loci that show a strong imprinting effect even when the overall effect of a locus is not significant (this leads to the inclusion of a single locus, *Wti3.1*, as a putative *i*QTL). When the locus shows significant parental imprinting, the contrasts generally identify the correct pattern of imprinting, ranging from 83% correct at the genome-level threshold when the locus accounts for just ½% of *Vp* to 99% correct when it accounts for 5% of *Vp*. In the rare cases where the contrasts identify the wrong pattern of imprinting, the incorrect patterns are evenly distributed between polar and bipolar dominance, but the locus almost never shows the alternative form of parental expression (i.e., if the locus is simulated to show maternal expression, the contrasts never indicate a best fit for paternal expression). When the locus shows bipolar dominance, power is generally higher than the case for parental expression and the contrasts also generally identify the correct pattern of effect of the locus. However, unlike parental expression, when the contrasts identify the incorrect pattern of imprinting, the locus shows polar dominance the majority of the time (⅔ to ¾ of the incorrect patterns are some form of polar dominance). This suggests that cases of polar and bipolar dominance may be somewhat confounded, but bipolar dominance very rarely appears as parental expression.

When the locus was simulated to show additive or dominance effects, imprinting effects were significant less than 5% of the time (i.e., less frequently than expected by chance using a 5% significance threshold; with the actual frequency being between 3 and 4%), indicating that non-imprinted patterns of genetic effect generally do not lead to the incorrect identification of a locus as showing imprinting. This result strongly suggests that a detected significant imprinting effect represents a case of true imprinting at a locus since the rate of false positives is lower than the rate expected under the null model. Furthermore, the fact that the model is very successful at identifying the correct pattern of effect of a locus (especially for loci accounting for ≥1% of the variance, as is the case for nearly all of the *i*QTL) provides strong support for the diversity of patterns of effect we describe for the detected *i*QTL.

## Supporting Information

Table S1 Additional information about the significant *i*QTL. Listed are the *i*QTL name, the traits affected by the *i*QTL, the marker name at the *i*QTL location along with its F_2_ map position in cM, and its physical coordinate in mouse genome build 36 (www.ensembl.org). The effect of the QTL is listed for all traits where the imprinting effect was significant. These are given as the additive (*a*), dominance (*d*) and imprinting (*i*) genotypic values, their standard errors (SE) and significance (*p*) value. These are followed by the LPR for the overall effect of a locus and the significance threshold at the chromosome level for the chromosome containing the QTL. Under the heading ‘Characterizing the pattern of imprinting’ are the ratio of the additive to the imprinting genotypic values (*a*/*i*), the dominance to imprinting ratio (*d*/*i*), the standardized imprinting values (*i*/SD), the standard deviation of the trait (SD), the *R 2* (rsq%) value of the imprinting effect alone and *R 2* for the overall variance explained by a locus, the best fit pattern along with the LPR value associated with the contrast for that pattern. These are followed by the genotypic values of the four ordered genotypes *LL*, *LS*, *SL*, *SS* and their standard errors (SE), which were estimated by the mixed model fitted by REML (i.e., the model used to detect and characterize QTL effects).
(0.08 MB XLS)

Table S2 Listed are the 353 SNP markers used in the study. The table includes the chromosome (Chr), marker name (Marker), F_2_ map position in cM [Map Pos (cM)] and their physical position based on mouse genome build 36 (ensembl.org) [Phy. Pos. (bp)].
(0.06 MB XLS)

Table S3 Power analysis of the mixed model to detect *i*QTL showing either parental or bipolar expression. The ‘*i*QTL’ column lists the pattern of effect simulated for a locus, ‘Family effect’ indicates whether the random effect of family was included in the model and ‘*%Vp*’ list the percent of phenotypic variance accounted for by the locus. These are followed by three pairs of columns that give the percent power and percent correct assignment of the real QTL effect pattern using a locus, chromosome and genome level significance test.
(0.10 MB DOC)
