# Supplementary material for: Genome-Wide Analysis Reveals a Complex Pattern of Genomic Imprinting in Mice
Source: PLoS Genet. 2008 Jun 6;4(6):e1000091. doi: 10.1371/journal.pgen.1000091 (PMC2390766; doi:10.1371/journal.pgen.1000091)
Supplement: Table S3 — Power analysis of the mixed model to detect iQTL showing either parental or bipolar expression. The ‘iQTL’ column lists the pattern of effect simulated for a locus, ‘Family effect’ indicates whether the random effect of family was included in the model and ‘%Vp’ list the percent of phenotypic variance accounted for by the locus. These are followed by three pairs of columns that give the percent power and percent correct assignment of the real QTL effect pattern using a locus, chromosome and genome level significance test. (0.10 MB DOC) [file pgen.1000091.s003.doc]

|  |  |  |  | Locus |  | Chromosome | | Genome | |
| --- | --- | --- | --- | --- | --- | --- | --- | --- | --- |
| *i*QTL | Family effect | % *Vp* | Test | Power % | Correct % | Power % | Correct % | Power % | Correct % |
| Parental | yes | ½ | Locus | 35 | 70 | 19 | 78 | 6 | 83 |
|  | no | ½ | Locus | 18 | 58 | 8 | 69 | 2 | 81 |
|  | yes | ½ | *i* | 42 | 65 | 16 | 59 | 4 | 57 |
|  | no | ½ | *i* | 25 | 49 | 8 | 41 | 1 | 50 |
|  | yes | 1 | Locus | 62 | 84 | 48 | 86 | 23 | 88 |
|  | no | 1 | Locus | 34 | 69 | 20 | 72 | 6 | 81 |
|  | yes | 1 | *i* | 66 | 82 | 37 | 78 | 11 | 69 |
|  | no | 1 | *i* | 40 | 63 | 17 | 52 | 2 | 26 |
|  | yes | 2 | Locus | 86 | 94 | 78 | 94 | 58 | 96 |
|  | no | 2 | Locus | 54 | 80 | 41 | 83 | 19 | 88 |
|  | yes | 2 | *i* | 87 | 93 | 65 | 92 | 32 | 91 |
|  | no | 2 | *i* | 59 | 76 | 32 | 74 | 7 | 59 |
|  | yes | 5 | Locus | 98 | 98 | 97 | 98 | 91 | 99 |
|  | no | 5 | Locus | 78 | 91 | 69 | 93 | 51 | 95 |
|  | yes | 5 | *i* | 98 | 98 | 90 | 98 | 70 | 98 |
|  | no | 5 | *i* | 80 | 90 | 58 | 90 | 26 | 89 |
|  |  |  |  |  |  |  |  |  |  |
| Bipolar | yes | ½ | Locus | 51 | 41 | 22 | 47 | 6 | 62 |
|  | no | ½ | Locus | 27 | 43 | 10 | 44 | 2 | 71 |
|  | yes | ½ | *i* | 69 | 41 | 41 | 50 | 13 | 67 |
|  | no | ½ | *i* | 43 | 48 | 18 | 59 | 4 | 89 |
|  | yes | 1 | Locus | 80 | 54 | 56 | 58 | 25 | 62 |
|  | no | 1 | Locus | 49 | 50 | 24 | 52 | 7 | 55 |
|  | yes | 1 | *i* | 91 | 53 | 73 | 59 | 40 | 70 |
|  | no | 1 | *i* | 64 | 50 | 38 | 61 | 10 | 70 |
|  | yes | 2 | Locus | 97 | 69 | 86 | 71 | 60 | 75 |
|  | no | 2 | Locus | 74 | 59 | 49 | 63 | 22 | 66 |
|  | yes | 2 | *i* | 98 | 69 | 95 | 70 | 78 | 76 |
|  | no | 2 | *i* | 86 | 59 | 65 | 66 | 28 | 78 |
|  | yes | 5 | Locus | 99 | 84 | 98 | 84 | 92 | 85 |
|  | no | 5 | Locus | 91 | 75 | 80 | 76 | 54 | 80 |
|  | yes | 5 | *i* | 100 | 83 | 99 | 84 | 97 | 85 |
|  | no | 5 | *i* | 96 | 74 | 88 | 77 | 63 | 84 |
